# Metabolic Insights Into the Effects of Nutrient Stress on *Lactobacillus plantarum* B21

**DOI:** 10.3389/fmolb.2019.00075

**Published:** 2019-08-30

**Authors:** Elvina Parlindungan, Bee K. May, Oliver A. H. Jones

**Affiliations:** ^1^School of Science, RMIT University, Melbourne, VIC, Australia; ^2^Australian Centre for Research on Separation Science, School of Science, RMIT University, Melbourne, VIC, Australia

**Keywords:** NMR, GC-MS, metabolomics, stress, lactic acid bacteria

## Abstract

*Lactobacillus plantarum* B21 is a strain of lactic acid bacteria first isolated from a fermented meat product from Vietnam. It is also a promising biopreservative with potential use in the food industry as it is a source of a novel bacteriocin (Plantacyclin B21AG) which has inhibitory effects against a wide range of species, including several pathogenic and spoilage strains. Nutrient stress is known to increase the survivability, storage stability, and bacteriocin production capability of *L. plantarum* B21 during industrial processing. It is however, unknown what the underlying biochemical responses that control these abilities are. This study therefore investigates the metabolite profiles of *L. plantarum* B21 using NMR spectroscopy and GC-MS to further understand the biochemical responses of this strain to various stress events. Unstressed cells were found to use glucose as their primary energy source with high concentrations of metabolites involved in glycolysis and organic acid synthesis, such as lactic acid, acetic acid, propanoic acid, malic acid, and 2-butenedioic acid being present in these cells. In contrast, large numbers of metabolites involved in amino acid metabolism including alanine, glutamic acid, aspartic acid, valine, proline, and norleucine were upregulated in glucose stressed cells, indicating that they were using amino acids as their main source of energy. Differences in levels of fatty acids, particularly octadecenoic acid, tetracosanoic acid, and 7-hexadecenoic acid were also observed between stressed and unstressed cells. The results from this study provide insight on the biochemical response of this bacterial strain to stresses commonly found during industrial processing.

## Introduction

Lactic acid bacteria (LAB) are of significant importance in the food industry due to their wide range of uses. These include starter cultures for fermented food products (Palomino et al., 2015), probiotics (Gómez et al., 2016), and as a source of natural biopreservative compounds (such as bacteriocins) to extend shelf life of food products by reducing spoilage (Behnam et al., 2015).

Food spoilage is a serious problem in the global supply chain. It has been estimated that a third of all food produced globally is lost or wasted, at a cost of more than $35 billion a year (FAO, 2011). A considerable share of these losses are caused by food spoiling due to microbial contamination during storage and transport. The use of chemical preservatives to control food spoilage, while well-established, has become increasingly unpopular with the public due to perceived health risks of such products (Amit et al., 2017). There is therefor, an increasing need to develop natural biopreservatives as an alternative. Bacteriocins from LAB have significant potential in this regard. The exploration of antimicrobial peptides has however, not yet been undertaken to any great extent even though some can control a wide range of pathogenic and spoilage bacteria. Currently, only one such compound, nisin, produced by *Lactococcus lactis*, has regulatory approval. This compound took ~30 years to be commercialized and has some significant limitations, in terms of its low solubility in water and the fact that it is not pH or temperature stable.

The LAB genus *Latobacillus* is generally considered safe for use in food products and *Lactobacillus plantarum* species have received Qualified Presumption of Safety (QPS) status from the European Food Safety Authority (EFSA) (Ricci et al., 2017). One particular strain of *L. plantarum* B21, first isolated from a traditional Vietnamese fermented meat product (nem chua), produces a novel, cyclic bacteriocin with good pH stability (pH 3.0–10.0), moderate thermostability (up to 20 min at 90°C), and high efficacy against a range of species (Tran, 2010; Golneshin et al., 2016). Most bacteriocins inhibit only closely related species, however, the *L. plantarum* B21 bacteriocin, Plantacyclin B21AG, is, like nisin, “broad-spectrum,” and effective against many Gram-positive organisms, including the lactic acid bacteria commonly associated with spoilage, and known pathogens such as *Listeria*.

For effective functional food applications bacteria need to survive harsh environmental conditions encountered during production and manufacturing. A significant reduction in cell viability may also occur during transport and storage (Sousa et al., 2012, 2015). Conversely, exposure of LAB to low/sublethal stress has been shown to improve long term stability, robustness, and viability (Serrazanetti et al., 2009; Ebrahimi et al., 2016). In the case of *L. plantarum* B21, we have previously shown that nutrient stress, and carbohydrate starvation in particular, results in alterations in morphology (Parlindungan et al., 2018) as well as improved storage stability and survivability and the retention of bacteriocin production capability (Parlindungan et al., 2019). It is however, unclear what the underlying biochemical responses to such stresses are that controlled these abilities are. In the present study therefore, nutrient stressed *L. plantarum* B21 were metabolically profiled through a combination of ^1^H Nuclear Magnetic Resonance (NMR) spectroscopy and Gas Chromatography Mass Spectrometry (GC-MS). The overall aim was to gain a more detailed understanding of the metabolism of this species as well as its biochemical responses to stress.

## Materials and Methods

### Bacteria Strain and Culture Storage

*L. plantarum* B21 were obtained from a culture maintained at the School of Science, RMIT University, Melbourne, Australia. The stock culture of this strain was stored at −80°C in De Man, Rogosa and Sharpe (MRS) broth (BD Biosciences, North Ryde, New South Wales, Australia) supplemented with 40% (v/v) glycerol (Sigma Aldrich, Castle Hill, New South Wells, Australia) prior to use.

### Preparation of Growth Media

Four different growth media were tested. The first was standard “MRS media” which was used as a positive control. This contained 10 g/L proteose peptone no. 3 (Oxoid, Scoresby, Victoria, Australia), 10 g/L lab-lemco powder (Oxoid), 5 g/L yeast extract (Sigma Aldrich), 20 g/L glucose (Merck, Bayswater, Victoria, Australia), 1 mL/L Tween 80 (The Melbourne Food Depot, Brunswick, Victoria, Australia), 2 g/L ammonium citrate (Sigma Aldrich), 5 g/L sodium acetate (Merck, Bayswater, Victoria, Australia), 0.1 g/L magnesium sulfate (Merck), 0.05 g/L manganese sulfate (Sigma Aldrich), and 2 g/L dipotassium phosphate (Merck). The second medium was termed “Tween 80 stress media” and was formulated as per the standard MRS described above but without Tween 80. The third medium was named “glucose stress media”; this was standard MRS medium without glucose. The fourth medium, “double stress media,” was formulated as standard MRS but this time without glucose and Tween 80. Glucose and Tween 80 were chosen as the main two stressors investigated in this study as it is known that both compounds are needed for high growth rates in *Lactobacilli* (De Man et al., 1960; Terraf et al., 2012).

### Preparation of Bacterial Pellet

*L. plantarum* B21 cultures stored at −80°C were transferred to MRS agar (Oxoid) plates and incubated for 24 h at 37°C. For each experiment three colonies were selected at random and transferred to one of the four different broth media described in section Preparation of Growth Media and incubated for 24 h at 37°C. A total of 0.1 g of bacterial pellet per broth type was then collected from each experiment by centrifugation at 8,000 × g for 10 min using an Eppendorf 5810R unit (Eppendorf, Hamburg, Germany). These pellets were then collected in eppendorf tubes (Sigma Aldrich) and each was washed three times with milliQ H_2_O for 5 min. These pellets were then used for metabolomics analysis via NMR and GC-MS. The viable cell count (CFU/mL) was ~10^9−10^ (for the unstressed control and Tween 80 stressed), ~10^8^ (glucose stressed), ~10^7^ (double-Tween 80 and glucose-stressed) as per Parlindungan et al. (2019). Each experiment was repeated three times (with three colonies) to make an *n* of nine for each treatment.

### NMR Sample Preparation

A standard methanol-water chloroform extraction was used to extract metabolites from the cells. A 2:1 mix of analytical grade methanol and chloroform (both Sigma Aldrich) was added to the bacterial pellets prepared in section Preparation of Bacterial Pellet and the mixture sonicated for 15 min. Analytical grade Chloroform and D_2_O (Sigma Aldrich) in a ratio of 1:1 were then added to form an emulsion. The sample was centrifuged at 8,000 × g for 20 min using an Eppendorf 5452 Minispin Centrifuge (Eppendorf). This generated distinct aqueous (upper) and organic (lower) fractions each containing different metabolites.

The aqueous layers were transferred to Eppendorf tubes (Sigma Aldrich) and evaporated to dryness using a TurboVap® LV evaporator supplied with N_2_ gas (Biotage, Rydalmere, New South Wales, Australia). The samples were then rehydrated in 500 μL of D_2_O in order to provide a deuterium lock for the NMR spectrometer. Since NMR shifts are sensitive to pH, 100 μL 0.24 M sodium phosphate buffer (PBS, pH 7) (Sigma Aldrich), was also added to each sample and a pH meter was used to ensure that samples had consistent pH of seven throughout the experiment. The PBS buffer also contained 1 mM of trimethylsilylpropanoic acid (TSP, Sigma Aldrich) as a reference standard to allow normalization of the resulting data.

### GC-MS Sample Preparation

The method used to prepare the samples for analysis via Gas Chromatography Mass Spectrometry (GC-MS) for this study was adapted from Jones and Hügel (2013). In contrast to the NMR, both aqueous and organic phase metabolites were analyzed.

#### Aqueous Fraction

After they had been analyzed via NMR each the aqueous phase sample was evaporated to dryness using the TurboVap® LV evaporator. A two-stage derivatisation reaction was then undertaken. For the first stage a 30 μL aliquot of methoxyamine hydrochloride (20 mg/mL in pyridine) (Sigma Aldrich) was added to each dry sample, which was then sealed, vortex mixed and left for 17 h. In the second stage, a 30 μL of N-methyl-N-trimethylsilyltrifluoroacetamide (MSTFA) (Sigma Aldrich) was added to the samples which were vortex mixed again for 30 s and then left to react for 1 h. The derivatized samples were then made up to a final volume of 600 μL with hexane prior to analysis via GC-MS.

#### Organic Fraction

The lipid content of the cell pellets was analyzed via GC-MS using the method of Atherton et al. (2008). The organic fractions from section Preparation of Bacterial Pellet were first left to evaporate in a fume cupboard overnight and then dissolved in 750 μL of a 1:1 mix of chloroform and methanol. Next, 125 μL of 10% BF_3_ in methanol was added and the samples incubated at 80°C for 90 min in an oven. The vials were left to cool for 5 min after which 300 μL of milliQ H_2_O and 600 μL hexane (Sigma Aldrich) were added before the samples were vortex-mixed. The aqueous (lower) layer was discarded and the organic (upper) layer was left to dry in a fumehood overnight. The dry samples were then reconstituted in 600 μL hexane prior to analysis via GC-MS.

### NMR Sample Acquisition and Analysis

NMR samples were analyzed using a Bruker-300 Ultrashield NMR (Bruker, Billerica, Massachusetts, USA) using 128 scans per sample. Free induction decays were multiplied by an exponential weighting function equivalent to 1 Hz line broadening after which they were Fourier transformed from the time to the frequency domain and referenced to the TSP single peak at 0.0 ppm. All spectra were then phased and baseline corrected manually. Areas of the spectrum <0 ppm and >10 ppm were excluded since they contained no data of interest. Prior to multivariate analysis the spectra were converted into numerical vectors, representing the individual metabolites, by integrating across the spectrum using 0.04 ppm integral regions (bins). The NMR spectral data were then converted to CSV format and imported into MetaboAnalyst 4.0 (Chong et al., 2018). The data was then normalized by sum and Pareto scaling was applied. A Principle Component analysis (PCA) scores plot, a biplot and box plots were generated and exported as TIFF files. Individual metabolites were identified using standards, our in-house library of peak shifts and with reference to chemical shifts detailed in the literature (Fan, 1996), NMR Suite Professional, version 8.3 (Chenomx, Edmonton, Alberta, Canada) and the Human Metabolome Database (HMDB) 4.0 (Wishart et al., 2018).

### GC-MS Sample Acquisition and Analysis

Samples were analyzed using a Clarus 680 GC-MS (Perkin Elmer, Wellesley, Massachusetts, USA) using splitless injection and a ZB-5MS column (30 m × 0.25 mm ID × 0.25 μm). Helium was used as the carrier gas at a constant flow rate of 1.2 mL/min. The GC oven temperature program was held at 70°C for 1 min, then increased at a rate of 10°C/min to 310°C and then held for a further 10 min. The MS was operated in electron ionization mode at 70 eV. Data acquisition was performed in full-scan mode from 50 to 650 m/z with a scan time of 3 s. The data were processed and analyzed using custom written software (“wsearch”) and metabolites identified using metabolite standards and with reference to the 2014 NIST mass spectral library. The absolute peak areas of identified metabolites were imported and converted to CSV format. The CSV files were then imported into MetaboAnalyst 4.0 (Chong et al., 2018). The data was normalized by sum and Pareto scaling was applied. A PCA scores plot, a biplot and a heatmap were generated and exported as TIFF files. A bar chart of the fatty acid data from the organic phase was generated using GraphPad Prism 8.02 (GraphPad, San Diego, California, USA).

### Statistical Analysis

To validate the PCA data, levels of each metabolite identified as significant from the NMR and GC-MS data were tested for significance using a one-way ANOVA verified with Tukey's *post hoc* test. A *p*-value of <0.05 was considered as statistically significant, with appropriate consideration given to samples with values close to this limit. All statistical analyses were performed using MetaboAnalyst 4.0 (Chong et al., 2018) and GraphPad Prism 8.02 (GraphPad). Unless otherwise stated, at least three independent experiments, each run in triplicate, were conducted (making an *n* of nine for each treatment condition) and the mean value reported.

## Results

### Metabolomic Profiling Using ^1^H NMR

**Figure 1:**
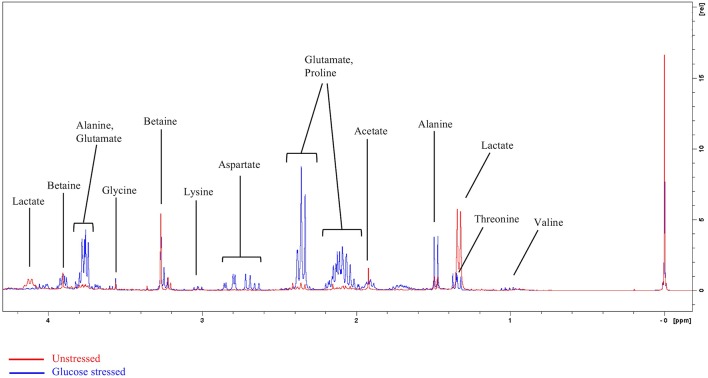
Representative overlayed ^1^H NMR spectra of glucose stressed (blue) and unstressed (red) *L. plantarum* B21. The peak at 0 ppm refers to the internal standard TSP.

^1^H NMR spectra of the unstressed (control) and glucose stressed *L. plantarum* B21 cultures are shown in Figure 1. In contrast to many metabolomics studies, clear differences can be seen by eye when comparing the spectra. For instance, lactate and acetate are present in high amounts in the unstressed control sample, but not in glucose stressed cell extracts.

**Figure 2:**
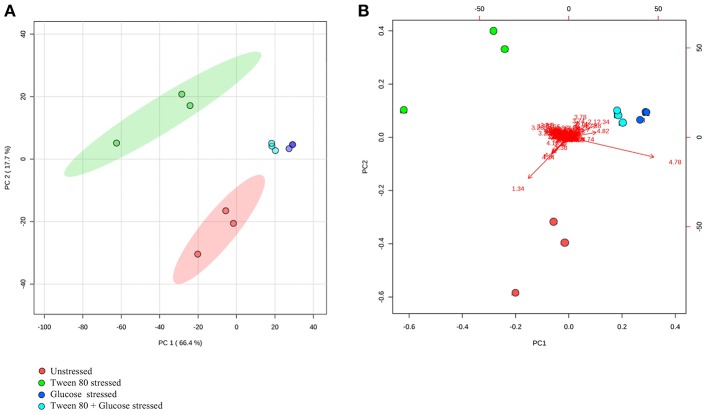
^1^H NMR metabolomic analysis of the unstressed control (red), Tween 80 stressed (green), glucose stressed (blue), and Tween 80 & glucose stressed (light blue) *L. plantarum* B21. Differences between control and stressed *L. plantarum* B21 were analyzed with **(A)** PCA score plot and **(B)** a biplot.

**Figure 3:**
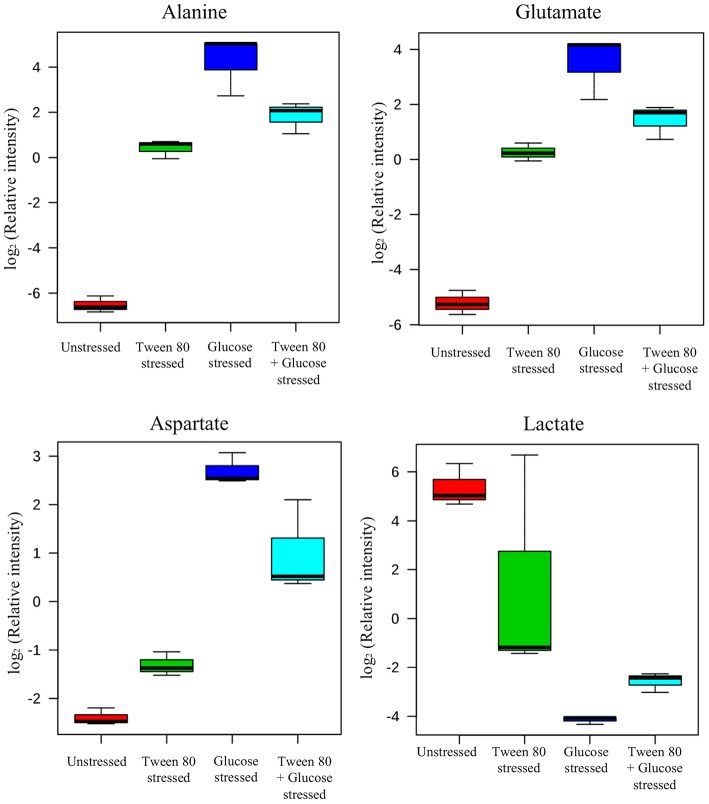
Box plots from ^1^H NMR data were generated. Identified metabolites, including alanine, aspartate, glutamate, arginine, and lactate were shown. Each box plot represents mean ± the standard deviation of the mean absolute peak areas from three independent experiments.

A clearer insight into the effect of glucose and/or Tween 80 stress on the metabolite profile of *L. plantarum* B21 was gained via multivariate analysis. The PCA score plot and biplot (Figures 2A,B) demonstrated clear variation in the metabolite profiles between the stressed groups and the controls (Figure 3). Alanine, glutamate and aspartate were significantly higher (*p* < 0.0005) in glucose stressed and double stressed cells, compared to unstressed and Tween 80 stressed cells. Lactate was found to be more abundant (*p* < 0.005) in the unstressed control and Tween 80 stressed cells but not in the other stressed groups.

### Metabolomic Profiling Using GC-MS

**Figure 4:**
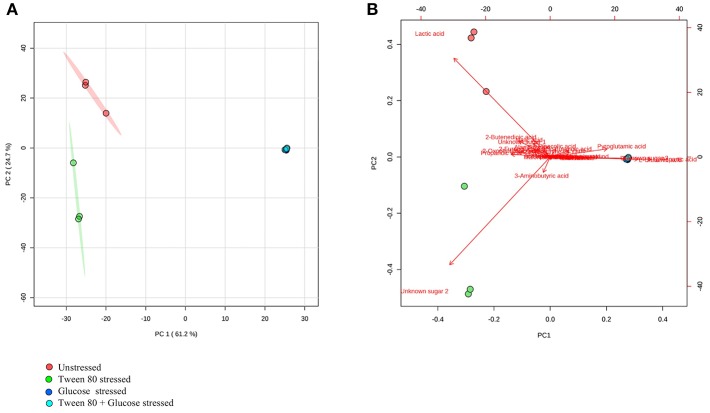
GC-MS metabolomics analysis of the unstressed control (red), Tween 80 stressed (green), glucose stressed (blue), and Tween 80 & glucose stressed (light blue) *L. plantarum* B21. **(A)** PCA score plot and **(B)** a biplot.

GC-MS based analysis also showed that there were clear and distinct metabolic differences between unstressed and stressed groups (Figures 4A,B). Lactic acid, 2-butenedioic acid and propanoic acid were high in the unstressed (control) group, while 3-aminobutyric acid and an unidentified sugar were higher in the Tween 80 stressed group. Glutamic acid and Aspartic acid and another unidentified sugar were found to be higher in both the glucose stressed and the double stressed groups.

**Figure 5:**
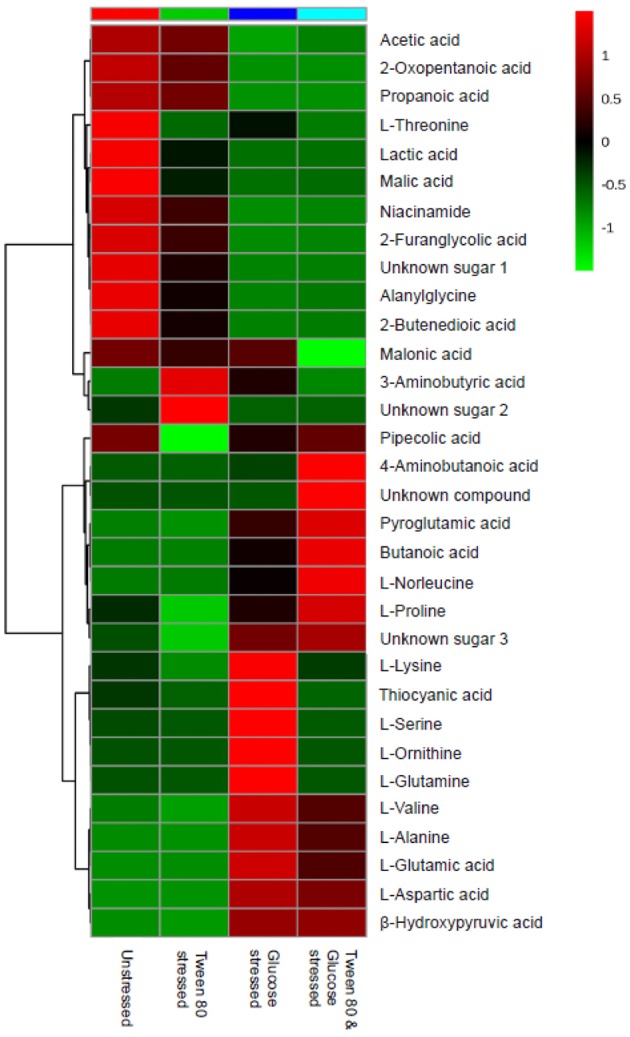
A heatmap from GC-MS data was generated to compare differences in metabolomics profile between the control and stressed *L. plantarum* B21.

A heatmap was generated to better display the metabolites causing separation between the control and stressed groups. A total of 33 metabolites were identified, including four compounds which could not be fully identified despite out best efforts (Figure 5). Acetic acid, 2-oxopentanoic acid, propionic acid, niacinamide, 2-furanglycolic acid, alanylglycine, 2-butenedioic acid, and an unknown sugar were highest in the unstressed control and Tween stressed groups, but these metabolites were absent in the other groups. Lactic acid, malic acid, an unknown sugar, alanylglycine and 2-butenedioic acid were significantly (*p* < 0.005) higher in the unstressed cells, whereas another unknown sugar was significantly (p < 0.005) higher in Tween 80 stressed group. A number of amino acids were found in glucose stressed and double stressed cells, but were absent in the other groups. These included norleucine, proline, valine, alanine, glutamic acid, and aspartic acid. Of these, lysine, glutamic acid and aspartic acid were significantly (*p* < 0.0005) upregulated in the glucose stressed cells compared to the double stressed cells. Some metabolites, such as 4-aminobutanoic acid, pyroglutamic acid, butanoic acid, L-norleucine, and L-proline were significantly (*p* < 0.005) more abundant in the double stressed cultures. In addition, thiocyanic acid, L-serine, L-ornithine, and L-glutamine were only expressed in the glucose stressed *L. plantarum* B21 cultures.

### Fatty Acid Profiling Using GC-MS

**Figure 6:**
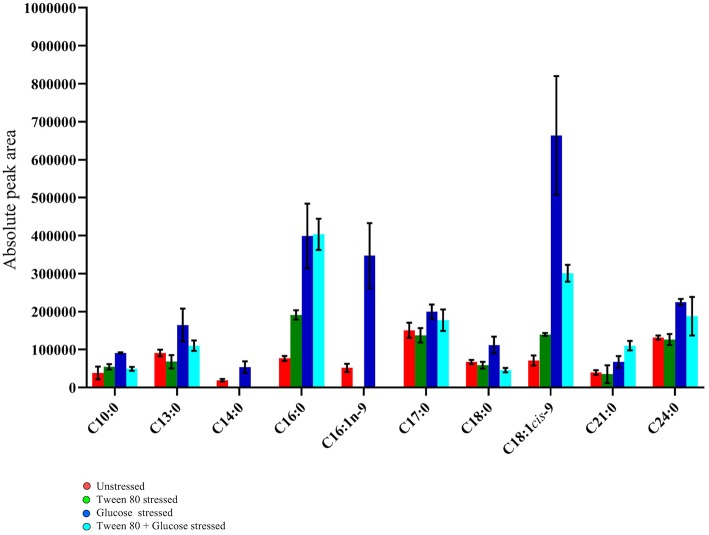
GC-MS fatty acid composition analysis of the unstressed control (red), Tween 80 stressed (green), glucose stressed (blue), and Tween 80 & glucose stressed (light blue) *L. plantarum* B21. Each bar plot represents mean ± the standard deviation of the mean absolute peak areas from three independent experiments.

Ten fatty acids (eight saturated and two unsaturated) were identified in total (Figure 6). The saturated fatty acids were decanoic acid (C10:0), tridecanoic acid (C13:0), tetradecanoic acid (C14:0), hexadecanoic acid (C16:0), heptadecanoic acid (C17:0), octadecanoic acid (C18:0), heneicosanoic acid (C21:0), and tetracosanoic acid (C24:0). The two unsaturated fatty acids detected were 7-hexadecenoic acid (C16:1n-9) and 9-octadecenoic acid (C18:1*cis*-9).

Tetracosanoic acid and 7-hexadecenoic acid were absent in Tween 80 stressed and double stressed samples. This may indicate that the absence of Tween 80 in the culture media inhibits the formation of these two fatty acids. Interestingly, 9-octadecenoic acid (*p* < 0.0005), hexadecanoic acid (*p* < 0.05) and tetracosanoic acid (*p* < 0.005) were highest in glucose stressed compared to Tween 80 stressed and unstressed cells. However, these fatty acids did not significantly (*p* > 0.05) differ between control and double stressed *L. plantarum* B21 cultures. Interestingly, the ratio of unsaturated fatty acids to saturated fatty acids varied between groups. The ratio was 1:3 in double stressed cells, 1:1 in glucose stressed cells, 1:5 in Tween 80 stressed cells and the unstressed control group. Overall there was higher percentage of unsaturated fatty acids produced when *L. plantarum* B21 was starved of glucose.

## Discussion

Bacteria encountering a sub-optimum environment may show behavioral or physiological adaptations to better cope with the stress. At a simple level such changes could mean expending additional energy on homeostasis or moving away from the source of the stress. Some organisms may also produce some form of chemical defense (e.g., bacteriocin) to try to reduce competition from other organisms. Under conditions of extreme stress, some species may decide to suspend growth and reproduction entirely until external conditions improve to give future offspring a greater chance of survival. *L. plantarum* B21 subject to starvation have been shown to produce more bacteriocin (most likely reduce competition for limited resources from other bacteria) and change their morphology to better survive the extreme conditions (Parlindungan et al., 2018, 2019).

**Figure 7:**
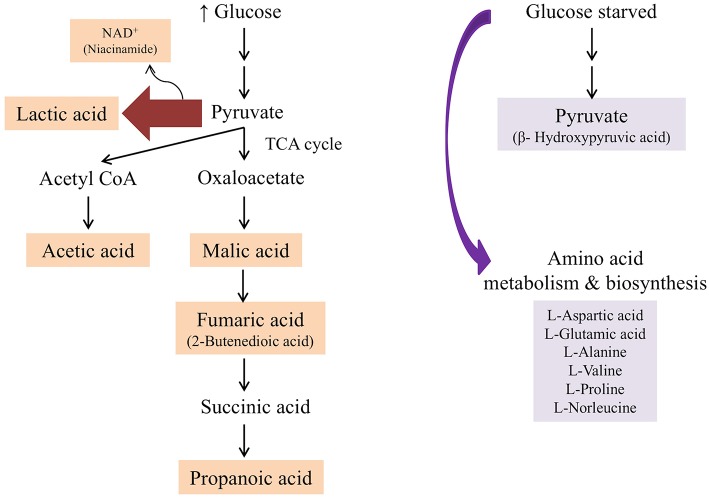
Summary of metabolic pathway conferred by *L. plantarum* B21 when grown in the presence of glucose (unstressed control and Tween 80 stressed groups) and when starved from glucose (glucose stressed and Tween 80 & glucose stressed groups) based on ^1^H NMR and GC-MS analysis.

In the presence of high glucose levels, bacteria commonly use a lot of energy for growth. LAB undergo fermentation in the presence of sugar with the predominant end product being lactic acid (Behera et al., 2018). A number of studies have also reported the production of a range of organic acids during fermentation in *Lactobacillus* species. These including acetic (Gobbetti et al., 2000; Zalán et al., 2009), formic, citric, butyric, and succinic acids (Zalán et al., 2009). *L. paracasei* CI6 has also been shown to catabolize valine into propanoic acid (Tammam et al., 2000). This could explain the high level of propanoic acid and downregulation of valine in unstressed and Tween 80 stressed *L. plantarum* B21 (Figures 5, 7). Downregulation of lysine, glutamic acid and aminobutyrate have previously been shown to be associated with the suppression of arginine deiminase (ADI), glutamate decarboxylases (GAD) and F_0_F_1_-ATPase proton pump pathways in *L. plantarum* ATCC 14917 (Wang et al., 2018). This could explain the downregulation of L-glutamic acid (Figures 3, 5), L-lysine (Figure 5), and 4-aminobutanoic acid (Figure 5) in the unstressed control and Tween 80 stressed groups observed in this study.

When LAB are grown in the absence of glucose, metabolic changes and adaptations to stress occur. The available energy within cells is utilized for protein and biomolecule synthesis rather than for generation of cell biomass. Proteins are used as a source of energy and degraded to generate peptides and amino acids (Thomas and Batt, 1968, 1969). It has also been reported that aminopeptidases cause protein turnover and new protein synthesis at transitional states during sugar stress conditions (Gottesman and Maurizi, 1992). This would explain the fact that a number of amino acids, including valine, alanine, glutamic acid, aspartic acid, proline, and norleucine, were upregulated in nutrient limited *L. plantarum* B21 (Figures 5, 7). High accumulation of β-hydroxypyruvic acid and the absence of lactic acid with other types of organic acids in the sugar starved *L. plantarum* B21 cultures also indicate inhibition of the fermentation and TCA cycles (Figures 5, 7).

Multiple studies have shown that in order to adapt to environmental stress, many bacteria alter metabolic pathways to conserve energy (Pages et al., 2007; Jozefczuk et al., 2010; Zhai et al., 2018). It has been argued that what might be termed the conserved energy metabolism mode can decrease intracellular reactive oxidative stress (ROS) levels and thus reduce ROS related damage (Zhai et al., 2018). The phenomenon of increasing survivability and functional resistance to stress has also been observed in *Vibrio vulnificus* and *Listeria monocytogenes* (Li et al., 2014; Highmore et al., 2018). Sugar starvation in *L. lactis* has also been shown to lead to the loss of phosphotransferase (PTS) sugar transport but not proton motive force (PMF) dependent metabolic pathways, or the ability to transport protein substrates via ATP (Ganesan et al., 2007). A switch to conserved energy metabolism mode (as demonstrated by the accumulation of amino acids) could be the reason for the high survivability and better storage stability (but retained functional bacteriocin activity) in spray dried, glucose stressed *L. plantarum* B21 in previous work from our group (Parlindungan et al., 2019).

Aside from shifting away from lactic acid production into amino acid metabolism/biosynthesis, stressed LAB may undergo alteration of their fatty acid profile (Ganesan et al., 2007). *L. plantarum* B21 has previously been shown to undergo morphological changes in response to nutrient stress (Parlindungan et al., 2018). In that study the absence of glucose in the growth media resulted in significantly shorter, coccoid-like cells, with ruffled cell surfaces, compared to control cells which were long and rod shaped, with a smooth surface. In the present study it was seen that this physical change is mirrored by a change in composition of fatty acids that may affect membrane permeability. Significant increases in unsaturated fatty acids were observed in the glucose stressed group (C16:1n-9 and C18:1*cis*-9) and in the double stressed group (C18:1*cis*-9) (Figure 6). A higher percentage of unsaturated fatty acids can improve the liquidity, flexibility and elasticity of cell membranes and promote extracellular H^+^ pumping and thus maintain intracellular pH homeostasis (Streit et al., 2008).

A decrease in fluidity of bacterial cell membranes has also been seen in response to other forms of stress. For example, the cadmium resistant strain *L. plantarum* CCFM8610 has a higher ratio of saturated fatty acids compared to the sensitive *L. plantarum* CCFM191 strain under normal, unstressed conditions. This may inhibit the entry of cadmium into the cytoplasm and thus provide protection from cadmium toxicity (Zhai et al., 2018). When these two strains were exposed to cadmium stress, *L. plantarum* CCFM191 showed more perturbations in its fatty acid profile compared to *L. plantarum* CCFM8610. Similar results were seen in O*enococcus oeni* 2219 exposed to phenolic stress compared to *L. plantarum* 2565 (Devi and Anu-Appaiah, 2018). *O. oeni* 2219 also showed a significant increase in the percentage of saturated fatty acids after exposure to phenolic stress but the opposite occurred to *L. plantarum* 2565. Higher ratios of unsaturated to saturated fatty acids have also been shown to contribute to acid resistance ability in *L. plantarum* ATCC 14917 (Wang et al., 2018).

When Tween 80 was absent in the growth media, the formation of tetradecanoic acid (C14:0) and cis-7 hexadecenoic acid (C16:1n-9) were inhibited in the Tween 80 and double stressed groups (Figure 6). A similar finding was made by Partanen et al. (2001) who assessed six strains of *L. delbrueckii* and found that all but one required exogenous Tween 80 or Tween 20 as a source for cellular unsaturated fatty acid biosynthesis. The presence of Tween 80 also led to an increase in the level of unsaturated fatty acids in *Streptococcus salivarius* ATCC 25975 (Jacques et al., 1985). Proteomic analysis by Al-Naseri et al. previously revealed that the presence of Tween 80 reduced the abundance of enzymes associated with fatty acid biosynthesis in *L. casei* GCRL163 (Al-Naseri et al., 2013). The researchers suggested the reason for the apparent effect of Tween 80 was due to the ability of LAB to directly incorporate the oleic acid part of the Tween 80 molecule into their cell membrane, which resulted in downregulation of *de novo* synthesis of fatty acids. However, given the variance in published data it seems likely that changes in fatty acid composition in response to stress is specific to both the form of stress and the strain of bacteria.

Cellular biomarkers are useful tools to predict bacterial robustness and survival in response to stress. *L. salivarius* FDB89 demonstrated significant increases in proline, isoleucine, phenylalanine, and methionine in response to hyper-osmotic stress (Qi et al., 2018). Significant increases in glycine and glutamate related metabolites were also previously observed in *L. plantarum* CCFM8610, where they were linked with tolerance toward cadmium, osmotic, and oxidative stress (Zhai et al., 2018). Other studies have reported increased expression of genes responsible for alanine incorporation into teichoic acids in response to bile and heat stress in *L. plantarum* WCFS1 (Bron et al., 2006).

It could be argued that the significant increased expression of glutamic acid, aspartic acid, alanine (Figures 3, 5), valine, glutamine, ornithine, serine, and lysine (Figure 5) revealed by the NMR and GC-MS metabolomics may be a potential biomarker of a sugar starvation stress response in *L. plantarum* B21. The upregulation of these compounds therefore could be the reason for high survivability and robustness in glucose stressed *L. plantarum* B21 (Bezkorovainy, 2001; Reid et al., 2007). The exact mechanism of bacteriocin activity in *L. plantarum* B21 could not be fully elucidated through this study however, and four compounds (Figure 5) could not be identified with GC-MS or NMR. Liquid chromatography (LC)-MS metabolomic and proteomic analysis could potentially be conducted to identify the unknown compounds and gain a deeper understanding on the impact of glucose and Tween 80 on bacteriocin production in *L. plantarum* B21. It is also is not yet known whether glucose stressed *L. plantarum* B21 are also resistant to other stressors encountered during manufacturing and processing such as heat or pressure.

Metabolomics is a powerful tool for phenotypic analysis to aid understanding global metabolite profiles in a biological system under a given set of conditions (Jones, 2018). Studying metabolomics stress response in LAB via metabolomics could also be useful to screen for useful compounds for food applications and industrial biotechnology. For example, lactic acid is a versatile organic acid extensively used in the cosmetic and pharmaceutical industries and for the production of polylactic acid (Gao et al., 2011; Zhang et al., 2014). Most of the world's commercial lactic acid is produced through modified or optimized microbial fermentation of *Lactobacillus* strains (Ghaffar et al., 2014). The worldwide demand for lactic acid reported to be roughly 130,000–150,000 tons per year and increasing rapidly (Wee et al., 2006; Farooq et al., 2012). In this study it was shown that *L. plantarum* B21 when grown in normal condition, without stress, produces a significant amount of lactic acid (Figures 3, 4B, 5). This strain of LAB could therefore, potentially be utilized for the mass production of lactic acid. Similarly, Tween 80 stressed *L. plantarum* produced a significant (*p* < 0.0005) amount of 3-aminobutyric acid (Figures 4B, 5) the esters and salts of which are currently used as flavoring agents in foods, as well as in cosmetics and pharmaceuticals (Liu et al., 2013). Similarly, glucose starvation in *L. plantarum* B21 resulted in upregulation of alanine, glutamic acid and aspartic acid (Figures 3, 5). Alanine is of great interest for use as a food sweetener and for pharmaceutical applications (Mollet et al., 2001; Papagianni, 2012). Meanwhile aspartic acid is an important compound for the production of aspartame (Prodolliet and Bruelhart, 1993). It is possible therefore, that a number of useful products could possibly be generated with LAB grown under the right culture conditions, but more work would be needed to prove this.

## Conclusion

In summary, ^1^H NMR and GC-MS based metabolomics showed that the stressed and unstressed *L. plantarum* B21 had very different metabolic profiles. High levels of organic acids including lactic acid, acetic acid, 2-butenedioic acid, malic acid, and propanoic acid were detected in control and Tween 80 stressed cells. In contrast, amino acids including alanine, glutamic acid, aspartic acid, valine, proline, and norleucine were the major compounds detected in glucose starved *L. plantarum* B21 indicating possible changes in energy metabolism. The higher percentage of unsaturated fatty acids in the glucose starved bacteria compared to controls could be an indicator of an increase in the cell membrane's fluidity in order to maintain cellular homeostasis. Using metabolomics to study the stress response of important microorganisms could also have significant importance in the food industry to generate specific compounds of interest.

## Data Availability

The datasets generated for this study are available on request to the corresponding author.

## Author Contributions

EP and OJ designed the study. BM assisted with the microbiology aspect of the work. EP conducted the experiments, analyzed data, and wrote the manuscript. OJ planed and supervised the experimental work, provided advice on data analysis, and co-wrote the manuscript.

### Conflict of Interest Statement

The authors declare that the research was conducted in the absence of any commercial or financial relationships that could be construed as a potential conflict of interest.
